# Biological computation: hearts and flytraps

**DOI:** 10.1007/s10867-021-09590-9

**Published:** 2022-01-28

**Authors:** Kay L. Kirkpatrick

**Affiliations:** Departments of Mathematics and Physics, University of Illinois at Urbana-Champaign, Illinois, USA

**Keywords:** Computation, Computational theory of mind, Biological computation, Biological information processing, Neural computation, Neurocardiology

## Abstract

The original computers were people using algorithms to get mathematical results such as rocket trajectories. After the invention of the digital computer, brains have been widely understood through analogies with computers and now artificial neural networks, which have strengths and drawbacks. We define and examine a new kind of computation better adapted to biological systems, called biological computation, a natural adaptation of mechanistic physical computation. Nervous systems are of course biological computers, and we focus on some edge cases of biological computing, hearts and flytraps. The heart has about the computing power of a slug, and much of its computing happens outside of its forty thousand neurons. The flytrap has about the computing power of a lobster ganglion. This account advances fundamental debates in neuroscience by illustrating ways that classical computability theory can miss complexities of biology. By this reframing of computation, we make way for resolving the disconnect between human and machine learning.

## Computability has been considered not active

The most celebrated biological computing system may be the brain, and it is widely believed to be performing various kinds of computations, but exactly which kinds of computations we do not fully understand. I argue here that although comparisons to digital computers are common, they are not particularly helpful for understanding the mysterious aspects of the brain, and thus we need a new concept, of a biological computing system as proposed here, which organically incorporates aspects of neural computation and of biological information processing that are outside of the notions of traditional computation and information processing, especially digital computation (see [1–3]).

Biological computing systems, defined in detail in the next sections, are diverse: some can perform digital computations, some can perform analog computations, and some can perform computations that are neither digital nor analog (see [4]). In addition, biological computing systems have processes that are not computational in any sense, and processes that intertwine with computational functions to augment the computational space in a way that digital computers and robots cannot do—and might not ever be able to do until we formulate new ways of making computational systems. There is hope that deep learning with artificial neural networks (ANNs) may be one such new way, but the theory is lacking for how such networks might or might not truly imitate human intellectual activity.

Among various accounts of computation, especially the excellent mechanistic account of physical computation which is useful for analyzing computing artifacts, computational functions are usually distinguished from non-computational functions [5]. In the context of biology, many non-computational functions are crucial for a biological system, including biochemical and mechanical functions. Thus I group these important non-computational functions together under the term “action,” a term chosen based on Alan Turing’s usage in his 1948 paper that distinguishes action from information processing and specifies four categories of machines [6]. Action will, however, turn out to include biological processes that also possess computational aspects.

Turing’s four categories of machines come from two dichotomies of machine type. His first dichotomy is continuous versus discrete type, and he says that while the brain may be continuous or analog, it can be modeled or approximated as discrete or digital. This insight has been used many times in neurobiological modeling.

His second dichotomy—between controlling and active type—turns out to be mistaken. Controlling machines, he writes, deal solely with information, and two of his examples are the telephone and the brain. (It should be noted that Turing’s use of the word “control” is different from its use in the cybernetics and control theory studies.) Active machines, on the other hand, produce definite physical effects that are larger than small random effects, and his example is a bulldozer. Turing does not specify, but it is reasonable to interpret his writing as saying that definite physical effects are significant effects that are also not Turing machine effects: erasing or writing digits, moving the head left or right, or changing the internal state.

To link this to mechanistic physical computation, it should be recognized that computation is not the same concept as information processing, but that processing information entails generic computation, although not necessarily digital computation [3, 5]. Thus we might be able to use evidence about how a system processes information as evidence for its doing generic computing, but not vice versa. It would be interesting to study the analogous relationship in the biological setting, where things ought to be different from the electrical and computer engineering setting, but subsequent sections of this paper will focus on biological computing.

Why is the active versus controlling dichotomy false? The brain does both processing of information and production of definite physical effects. The brain’s physical effects include making chemicals such as hormones and neuromodulators, making movements mediated by muscles, physically remodeling its own structure in processes such as learning and neuroplasticity, and generating electromagnetic fields. (As examples of significant electromagnetic fields, electric fish use neural tissue to generate electrical waves that stun or steer their prey, and Rodrigo Hübner Mendes can drive a racecar with no limb movement but instead with an EEG helmet developed by Tan Le [7].) We summarize these definite physical effects and others like them by the term *action*, similar but not identical to the term behavior.

Turing’s kind of dichotomous thinking has been common for decades and has limited how we think about the brain and the mind [8, 9]. It is common to invoke Shannon’s theory of communication to describe the brain as purely processing information, an approach shared by Turing, von Neumann, and others who have viewed the digital computer as the best metaphor of the brain’s activity. But restricting attention to information going into and coming out of the brain elides the complexity of the brain’s activity. This is appreciated by neuroscientists and computer scientists who work in subfields such as situated and embodied cognition [10].

The mechanistic account of physical computation comes close to articulating the particular abilities of biological computing systems, after some subtle modifications into a new account which we will call “biological computation.” This will be closer to a new model of computation that encompasses both action and traditional computation.

## Toward a more expansive kind of computation

Why do we need a new account that we call “biological computation” rather than simply applying the mechanistic account of physical computation to biology? There are several reasons.

First, in a typical biological system, there is no clean separation between physical action and computation, because these two processes can be intertwined in spacetime, coincident in space and simultaneous in time. A good account of biological computation must consider action and computation being intertwined like this. (We use “biological” to mean related to living things, including all cell-based life, although the exact boundary between life and organic non-life will not be at issue here.)

Second, biological systems have limitations compared to medium-independence in general physical computing systems as defined in [5], and these limitations must be accounted for in a new notion that we will call *medium flexibility*, defined in the next section. Medium-independence is a property of a computational vehicle (often a string of digits) for which the input–output rule that defines a computation is “sensitive only to differences between portions of the vehicles along specific dimensions of variation–it is *in*sensitive to any other physical properties of the vehicles” ([5], p. 122). These rules can be implemented in “multiple physical media (e.g., mechanical, electro-mechanical, electronic, magnetic, etc.), provided that the media possess a sufficient number of dimensions of variation (or degrees of freedom) that can be appropriately accessed and manipulated and that the components of the mechanism are functionally organized in the appropriate way.” We need a stricter notion of medium flexibility to account for the constraints of biology, and this stricter notion may allow us to demonstrate that biological computing systems may have better features than digital computing systems. (It is common in mathematics that stricter assumptions lead to theorems with better conclusions.)

Third, we need to understand the similarities and the differences between digital computers and biological computers if we want to eventually build devices that imitate humans or animals. It may be obvious already that there is a gap between the capabilities of digital computers and biological computers, but clarifying exactly what the differences are and formulating a new precise account of mechanistic biological computation may be essential to bridging the gap.

The first two reasons above, for why we need an account of biological computation specifically, demonstrate the two main modifications to the mechanistic account of physical computation we will make in order to arrive at the new account of mechanistic biological computation presented below: the medium-independence adapted into medium flexibility; and the explicit addition of possible action within and interaction with the surroundings. Both of these modifications are natural extensions of mechanistic physical computation considering the specific capabilities of biological systems, and they are both consistent with the general approach of *ceteris paribus* laws.

This new account of mechanistic biological computation should shed light on the most prominent biological computing system that we would like to understand: the brain. But a detailed deeper understanding of the brain as a biological computing system will have to be laid out in future work, partly because many functions of the brain are still poorly understood by neuroscientists. In the present work, we focus on a few examples of biological computing systems that are well-understood and may be considered “edge cases” of computation. A closer look at these systems, however, reveals that they are not mere mechanical machines but rather that they perform specialized computations and actions—which should be considered together as parts of the biological computing system. Some examples of biological computation do not involve neurons, which is an important takeaway point: neurons are not the only building blocks for biological computation, although they may be important building blocks for cognition.

### An account of biological computing

Roughly speaking, a biological computer is a living organism or one of its systems or components that can perform computational functions. More precisely, a biological computing system is a biological mechanism or system whose teleological function is to do *biological computation*, namely the manipulation of medium-flexible biological vehicles according to general rules that are sensitive only to biologically distinguishable differences between different spatiotemporal portions of the vehicles. Properly speaking, biological computing is a feature of a model for a biological system, and it could be that one model of a given system is a biological computer and another model is not. Models have the advantage of being mathematically concrete for checking whether the different parts of the definition below are satisfied, and the use of models explicitly acknowledges where our scientific understanding is limited, but informally we may identify the system with its model in the following sections. In the rest of this section, we make precise the terms used above: biological vehicles, biological particles, medium flexibility, manipulations, general rules with conditional branching and output actions, and biologically distinguishable differences; then we discuss a few anchoring examples before discussing the heart as a biological computer.

A *(biological) vehicle* is a biological and physical variable defined by physical degrees of freedom and by biologically relevant features such as specific biochemical composition. It can be 1) a variable that can take different values and change over time or from system to system, or 2) a specific value of such a variable. Biological variables often have many degrees of freedom, or even infinitely many. A biological vehicle may be reducible to a string of digits in some cases or, if the vehicle has continuous aspects that cannot be discretized reasonably, it may need to be described as a collection of particles with their individual degrees of freedom, usually position and momentum. Sometimes, notably at the scale of atoms and small molecules, biological vehicles may involve random variables or stochastic processes, but this is often an unnecessary complication at the scale of cells or tissues.

A *digit* as usual is a portion of a vehicle that can take one of finitely many states during a finite time interval, e.g., a binary digit, or bit. But because most biological systems also have non-finite and non-discrete aspects, we will also define a *(biological) particle* as a portion of a vehicle that can take one of possibly infinitely many states (positions and velocities, for instance). It is sometimes the case that only finitely many states are biologically distinguishable or have biologically distinguishable differences, so particles are a generalization of digits. Biological vehicles can be either abstract, as in “the bloodstream,” or concrete, as in “this particular spatiotemporal configuration of particles as a particular portion of a particular creature’s bloodstream at a particular time.” Another abstract vehicle is “an electrical wave” or “the action potential” and a related concrete vehicle may be “the particular electrical wave caused by particular ions’ movements through a particular organism’s tissue at a particular time.”

We require biological vehicles to be *medium-flexible*, a more constrained account than medium-independence [5, 11]. In order to be medium-flexible, a concrete biological vehicle is partially independent of its physical implementation, i.e., there are a number of possible biochemical compositions of the vehicle that can yield the same or similar behavior, but there’s a narrower range of options than for digital computers, typically. A vehicle is medium-flexible if and only if a general rule (defined below) is sensitive only to biologically distinguishable differences (which depend on context and are explained more at the end of this section) between parts of the vehicle along biological dimensions of variation and is insensitive to other physical properties of the vehicle that are not biologically distinguishable. A given biological computation can be implemented in multiple media provided that they have enough degrees of freedom and the right kinds of variability that can be appropriately accessed and manipulated, and provided that their components are functionally organized in the appropriate way.

As an example, blood is medium-flexible: different individuals and different species have different exact compositions of their blood, with different blood proteins, lipoproteins, and hormones. Two different species’ proteins may be homologous but not identical, even if they have more-or-less the same function, which is why we call these alternate implementations “flexible” but not “independent.” Unqualified medium-independence does not seem to be possible for blood: there appear to be more restrictions on what could be processed through the cardiovascular system without harm than, for instance, what media a user might use to consume a book on a laptop, assuming appropriate compatibility and software support. It is likely, however, that medium-flexible biological vehicles are locally medium-independent, in the sense that they could be simulated by other media if one were to focus on a small enough region of time or space. But biological vehicles must be considered globally in addition to locally, and the example of blood transfusion illustrates why medium-flexible is essential for biological computation. Transfusing blood among humans is asymmetric because of blood types, and transfusing the blood of one species into another species is almost surely disastrous for the recipient. (We also think of the bloodstream vehicle as hardware and software simultaneously, an occurrence in biology that is so common that it could be called “ambiware.”)

Continuing the account of biological computing, we also require the *manipulation* of the biological vehicle to be a transformation of one portion of a vehicle into another, with a possible simultaneous change in the system. As an example of the manipulation of an electrical wave in a tissue, we can consider a cardiac muscle cell receiving a wave of ions through gap junctions and passing the wave onward through other gap junctions while simultaneously contracting its muscle fibers. This example of manipulation illustrates the possibility that actions taken by the system can be simultaneous with the transformation of the vehicle, both of which will be included in the definition of general rule for the biological context.

A *general rule* is a map from inputs and internal states to (computational) outputs, (biologically relevant) activity outputs, and internal states. In mathematical notation, the rule is a well-defined function:

$$g: I \times S \rightarrow O \times A \times S,$$

where *I* is the set of inputs, *S* the set of internal states, *O* the set of traditional (computational) outputs in the sense of Turing machine effects (erasing/writing digits, moving the head left/right, and changing internal state), and *A* the set of output *actions* that are physical effects but not Turing-machine effects. We can construct the sets *O* and *A* by putting actions that transform a portion of a vehicle into another portion of the vehicle into computational outputs *O*, and putting actions that change something besides the vehicle into activity outputs *A*, e.g., physical movements inside the system or within its environment.

In examining whether a particular system or model does biological computing, a potential general rule is a mathematical relation: a subset of the cross-product of domain and range. In order for this general rule to correspond to a biological computation, it must be well-defined (single-valued and unambiguous). The general rule may be given by an algorithm or state transition table, but it need not be. It needs to have some conditional branching in order to be considered a nontrivial computation; alternatively and equivalently, some sort of while loop. A ribosome is an example which does biological computation in the present sense and which has been understood to be doing a kind of Turing computation in the cellular context [12]. In the following sections describing the general rules used by the heart and the flytrap, portions of the general rules can usually be described by an “if ... then ...” conditional structure.

A *biologically distinguishable difference* in a vehicle means that the biological system can distinguish different particles, for instance by having a receptor that binds one type of particle but not another type. Within a single species, say humans, there can be significant differences such as blood types, and these differences can be asymmetric: a person with O negative blood type can donate to a person with any blood type, but a person with A negative blood type cannot donate to the O negative person.

Smaller molecules like hormones can also have biologically distinguishable structures among different species. For example, the horse estrogen equilin differs slightly from the human estrogen estrone by having one less hydrogen atom. This is a biologically distinguishable difference even though it seems small, because it can cause biological effects for humans who take horse-derived hormone therapy, effects that are different from endogenously-produced human estrogen and effects that include altered liver function.

Even a subatomic difference such as one more neutron may or may not cause detectable differences in biochemical reactions, depending on context. Molecules with different isotopes are processed similarly in biochemical reactions, but often the heavier isotopes react more slowly, which may or may not matter macroscopically to the organism, depending on the reaction in question and the biological function.

Medium flexibility and biologically distinguishable differences are closely linked to each other, and both are properties of biological vehicles and, importantly, of biological systems or their descriptions. For some descriptions, especially descriptions at different scales, specific inputs are not biologically distinguishable, and medium flexibility can account for these indistinguishable inputs being mapped to the same output in one context, even if in another context they may be mapped to different outputs. For example, at the microscopic scale of iodide transport in the thyroid, the transporter proteins cannot distinguish between iodide and thiocyanate and allow both into the cells. On the macroscopic scale of the organism over a long time, however, thiocyanate is toxic. Context matters here.

As another example, the heart is medium-flexible, which can be illustrated by fish and frogs having two- and three-chambered hearts, and by artificial hearts being used to take over blood pumping for a human heart to some extent. Limitations of engineering have meant that artificial hearts are only a short-term solution as a bridge to transplantation, because they are not sufficiently similar to organic hearts, causing problems such as blood clotting. Similarly, same-species organ transplants are possible and illustrate medium flexibility and also limitations that may prevent full medium-independence, such as organ transplant recipients needing to take immuno-suppressants to prevent rejection of the transplanted organ. And of course, hearts vary widely across species. The turtle’s heart has three chambers with a partial wall that approximates warm-blooded animals’ four chambers. The blue whale’s heart is the size of a refrigerator, weighs about 400 pounds, and beats 8 to 10 times per minute. These variations and limitations illustrate some intricacies of medium-flexible systems: parts may be replaceable or implementable by other media, but only within constraints that are necessary for functioning well enough to sustain life.

#### A note on bio computational complexity

This account of biological computation can support a notion of biological computational complexity, which would be useful for making distinctions between biological computing systems that are simple and ones that are more sophisticated. One way biological computational complexity might be formulated is by borrowing from electrical and computer engineering and by defining something analogous to circuit complexity, which roughly measures the number of gates. A likely analogue of a gate in engineering is a molecular switch in biology, and there may be other structures playing similar roles as well. With such a complexity measure, we can use thresholds: if a biological system has a complexity that is less than a low threshold then we consider it trivial, and if other systems have complexities that are above higher thresholds then we consider them increasingly sophisticated. One hope is that systems satisfying higher thresholds could be proved to have special properties that systems below a low threshold do not or cannot have.

#### How biological computing stacks up

This account of biological computation is designed for the particular complexities of living systems, including those that are not accounted for in computability theory. Medium flexibility is also meant to follow nature’s lead and is likely to be somewhat between medium independence and multiple realizability: parts of biological computing systems might be medium independent on some times-cales or length-scales but not on others, and portions of a biological vehicle may be medium independent in some parts of the system but not in others. Here are some examples and non-examples of biological computing.

The first non-example, a biological process that does not do biological computing, is pure transport that disregards the degrees of freedom of the vehicle or objects being transported. This is because the rule that would describe this process is ill-defined or has no conditional branching. (The term pure is used here to mean isolated from other aspects of the system.) A special case is pure fluid flow, again, in isolation. Specifically, a model of fluid flow through the interior of a short segment of a (say, blood) vessel for a short period of time is non-computational, and if the flow is turbulent then a purported general rule could be ill-defined, and in any case the rule does not have conditional branching that is sensitive to biological degrees of freedom.

By contrast, directed transport–such as that done by signal recognition particles in the cell for getting proteins to the right location based on signal peptides–should be a positive example of a biological computation with perhaps approximately the computational complexity of a few switches or gates. Roughly speaking, a conditional branch in the general rule may correspond to a switch of some sort in the system.

Biological activities that appear similar to each other but happen on different scales may have dissimilar computational properties. For example, ionic flow down a gradient is not itself a computation, although a protein actively gating the ions might be performing a simple computation. (For the ionotropic NMDA receptor, both glycine and glutamate must bind for the ion channel to open, which makes it similar to an AND gate. Moreover, the presence of zinc, magnesium, or pharmaceutical compounds can modulate its function up to anesthesia.) But E. coli going up a sugar gradient is an example of biological computation, because it requires the coordination of a network of sensory and motor proteins, analogized to a neural network [13, 14]. Similarly, artificial neural networks (ANNs) should correspond to relatively simple biological computing systems, although ANNs often lack the active component that I believe is a hallmark of biological computing, and ANNs are not particularly accurate models of networks of actual neurons.

Another special case of transport is the process of an organism falling. In the absence of wings or similar controlling mechanisms, the trajectory of the organism and the forces of impact could be computed by an observer, but the organism itself does no computation; it just lets the process happen on the short time-scales of the fall and the impact, including tissue compression and possible damage. On very long time-scales, of course, certain species have evolved adaptations to reduce the damage from the falls that they are likely to experience.

More non-examples are pure random processes, at least when not part of a non-deterministic computation. For instance, a chromosomal crossover in meiosis is non-computational in isolation, although many such crossovers are necessary but not sufficient parts of variation-and-selection algorithms that we call evolution.

Many of these non-examples share the feature that if one were to try to write down the general rule *g* that they are following, one would arrive at a multi-valued or ill-defined function instead of a single-valued well-defined function. (As an aside, the multi-valued case includes certain stochastic biological processes that are actually computational and analogous to randomized algorithms instead of deterministic ones.)

Stepping back, biological cognition and neural computation are special kinds of biological computation, and a better understanding of the complexities of biological computation may help us zero in on the particular specialities of nervous systems. This framework could also be extended to computing systems involving material constraints that are not necessarily biological. Medium flexibility in a technological setting should describe aspects that traditional Turing computation cannot, such as human–computer interactions, row-hammer hacking (accessing registers nearby a target register and then doing synchronized bit-flipping to make an electromagnetic field that flips a bit in the target register), and electronic design constraints due to quantum tunneling, much like the constraints in biology.

## The heart as a biological computing system

The heart is a biological computing system that is similar in sophistication to a calculator, as argued below, having analog and digital computation functions as well as active, e.g., mechanical, functions. We begin by describing some of the vehicles for the heart as a biological computing system, and then describe a variety of control functions both internal and external to the heart.

The main vehicles of the heart (or the cardiovascular system) are the bloodstream (which can be subdivided into portions as described below), the internal electrical activity governed by auto-rhythmic nodal cells, and electrical activity generated by neurons in the heart’s ganglionic plexa. In addition to those vehicles in the cardiovascular system, there are adjacent control functions (and vehicles in their own right), the electrical and chemical activities of neurons whose cell bodies are outside of the heart that have axon projections onto various heart cells.

These vehicles are inherently complicated, but can be described in a set of ways framed nicely in terms of statistical mechanics. Statistical mechanics embraces microscopic and macroscopic descriptions simultaneously: microscopic variables are often high-dimensional and can be specified to varying degrees of accuracy and explicitness, and macroscopic variables may summarize the microscopic details or measure the quantities used in medicine for diagnosis and treatment. Importantly, which variables count as macroscopic depends on context in human biology, even within the same system, which is a feature of medium flexibility.

### The bloodstream is a medium-flexible vehicle

This section has a few detailed descriptions of the microscopic variables (the large vectors below) and then connects those to macroscopic variables such as stroke time (T), end-diastolic volume (EDV), stroke volume (SV), heart rate (HR), and heart rate variability (HRV). These macroscopic variables (macrostates for short) change in time and will have subscripts to keep track of that. Notably, HRV is an important measure of heart health, and it can be extracted from a reasonably large sequence of stroke times or inter-stroke intervals, though we will not get into that here. Two of the macroscopic variables, HR and HRV, are tightly linked to the electrical activity of the heart that is described in a later section.

All of the macroscopic variables listed so far are relevant to the activity in the interior of the heart’s chambers; other macrostates matter elsewhere in the heart, such as the epinephrine concentration in the heart’s own blood supply, and microstates matter in capillaries. The fact that different variables are salient in different parts of the system is part of what the notion of medium flexibility can capture. Next are some natural ways to describe the bloodstream as a vehicle and to divide it into portions, together with salient properties of these portions and specific ways that the portions of this biological vehicle can be manipulated by the heart.

The whole bloodstream can be described by a sequence of variables (functions, generally, and in a bit we will consider vectors) chosen by the natural divisions of the spatially separate left side ($\ell$) and right side (*r*) of the heart and the temporally sequential systolic contractions and diastolic fillings that it goes through: $\{ (E_{i,r}, S_{i,r}, T_{i,r}, E_{i,\ell }, S_{i,\ell }, T_{i,\ell }): i \in \mathbb {Z}\}$. Here $E_{i,r}$ is the end-diastolic (end-filling-of-ventricle) contents of the right side of the heart at the *i*th stroke or heartbeat, $S_{i,r}$ is the contents of the stroke volume ejected from the right ventricle with the *i*th stroke, and $T_{i,r}$ is the time at which the *i*th stroke occurs. Similarly, the variables with $\ell$ subscripts name the analogous things on the left side of the heart. Usually $T_{i,r} = T_{i,\ell }$, in which case we could write them both as $T_i$, and the other case is when an individual has a split S2, the medical term for the aortic and pulmonary valves closing sequentially instead of simultaneously.

A portion of the bloodstream vehicle, say $S_{i,\ell }$, which is the contents expelled by the *i*th stroke of the left ventricle, can be represented in several ways. An accurate and detailed way to represent it is by a time-dependent vector $\mathbf {V}_{i,\ell }(t) = (\mathbf {V}^{(j)}_{i,\ell }(t))_{j=1}^{N}$, where *N* is the number of particles in $S_{i,\ell }$. Each particle is tracked by its position and velocity in three dimensions as a function of time: for each *t* we write the *j*th particle’s state vector as $\mathbf {V}^{(j)}_{i,\ell }(t) \in \mathbb {R}^{6}$. The full vector $\mathbf {V}_{i,\ell }(t)$ is 6*N*-dimensional, and the entries are real-valued functions of time *t*.

Working with this full vector of functions is of course not practical, so we can discretize the full detailed vector $\mathbf {V}_{i,\ell }$ to a reduced vector $\mathbf {R}_{i,\ell } = (R_{i,\ell }^{(m)})_{m=1}^{M}$, where *M* is the number of distinct particle types, and entries track the number only of each particle type and not their every location. The *m*th entry $R_{i,\ell }^{(m)}$ is defined to be the number of particles of type *m* in the vehicle portion $S_{i,\ell }$. Then this reduced vector is *M*-dimensional, much lower dimensional than the 6*N* dimensions of $\mathbf {V}_{i,\ell }$ above. Concentrations of particles such as those measured in blood tests correspond to normalizing the entries of this reduced vector.

The end-diastolic portion $E_{i,\ell }$ can be described by similar full and reduced vectors, which are unnamed here because of notational limitations; the reduced vector for $E_{i,\ell }$ has entries with values that are approximately double the entries in $\mathbf {R}_{i,\ell }$ because of the portions’ relative volumes: a typical end-diastolic (full ventricle) volume is around 140 mL, and a typical stroke volume is around 70 mL of blood ejected before the aortic valve closes. (Ejection fraction is the ratio of the stroke volume to the end-diastolic volume, commonly around 50 or 60%.)

These vehicle vector variables can be further reduced in dimension to variables in standard medical terminology as follows. The end-diastolic volume of the *i*th stroke on the left side can be written as $EDV_{i,\ell }$ and is equal to the volume of the bloodstream portion $E_{i,\ell }$. Similarly $EDV_{i,r}$ is the volume of the right-side portion $E_{i,r}$. The stroke volume of the *i*th stroke on the left side is $SV_{i,\ell }$ and on the right side is $SV_{i,r}$. Note that the equality of left- and right-side stroke volumes, $SV_{i,\ell }=SV_{i,r}$, is usually true for healthy hearts, and thus that single value can be written as $SV_i$ for the *i*th stroke. (If the two sides’ stroke volumes are not equal, as in one-sided heart failure, then this creates a backup of blood in the flow approaching the failing side of the heart. Symptoms include swelling in the lungs and shortness of breath in right-sided heart failure, and swelling in the legs in left-sided heart failure.) Another standard medical variable is the heart rate, which we can identify at the *i*th stroke as the inverse of the time lapsed between the previous stroke at $T_{i-1}$ and the current stroke at $T_i$, that is, $HR_i = 1/(T_i-T_{i-1})$.

The volume variables $EDV_{i,*}$ and $SV_{i}$ are basically real-valued, or continuous, and the heart rate variables $HR_i$ are also real-valued if time is continuous, although they can all be approximated by discrete variables.

The previous *M*-dimensional vector variables, such as $\mathbf {R}_{i,\ell }$, allow keeping track of extra data beyond the standard medical variables: not only the volumes of those portions of the vehicle, but also their contents, which becomes important in the coronary part of the circulatory system and in the rest of the circulatory system to account for inputs and outputs. The portion of blood $S_{i,\ell }$ leaving the left ventricle at a particular stroke can also be subdivided into a small portion that goes into the coronary circulation and the remaining larger portion that goes into the body’s systemic circulation, so that the contents of that stroke can be written as the disjoint union of the coronary portion contents and the systemic portion contents. By contrast, on the right side of the heart, the entirety of the blood portion $S_{i,r}$ leaving the right ventricle goes into the pulmonary artery and into the lungs.

For an example of the interplay between the (macroscopic) medical data and the extra (microscopic) data, consider an interval of about a minute around the time when epinephrine is released by the adrenal glands and influences the heart to increase its output: say the zeroth stroke is the time $T_0$ when epinephrine is released, the tenth stroke at $T_{10}$ when the epinephrine-rich blood is passing through the right ventricle, the 30th stroke at $T_{30}$ when the epinephrine-rich blood returns from the lungs to the left ventricle, and the 40th stroke at $T_{40}$ when epinephrine reaches the myocardial cells in the coronary circulation and starts binding to cell-surface adrenergic receptors.

At the time $T_0$ the heart rate may be close to resting, around 70 beats per minute, so the inter-stroke intervals $T_i-T_{i-1}$ are each about 860 milliseconds, and the stroke volumes $SV_i$ are around 70 mL. Around half a minute later when epinephrine starts binding receptors at $T_{40}$, it takes some time for the receptors to reach saturation, say at $T_{50}$, and during this interval it is the case that myocardial cells are ramping up their glucose supply in response to the bound epinephrine, aiding the increase of both heart rate and the contractile strength of the heart muscle cells. By the end of this ramp-up, the heart rate may be up to around 140 beats per minute, and the stroke volume $SV_i$ up to around 100mL. Thus the stroke volumes have increased from time zero, $SV_{50} > SV_0$, and the inter-stroke intervals have decreased, $T_{50}-T_{49} < T_1-T_0$, inequalities that correspond both to biologically detectable differences and to changes in the medical data. Additionally, the detailed data about the contents of the bloodstream portions $S_{i,\ell }$ exhibit biologically detectable differences, e.g., the left-chamber contents of the earliest strokes, $S_{0,\ell }$, has a low level of epinephrine (say 0 picograms per mL) and the left-chamber contents of the 30th stroke, $S_{30,\ell }$, has a high level (say 140 picograms per mL), and later on the epinephrine level will have decreased back to basically zero after binding and breaking down.

How well a heart can adapt to changes, such as these changes in epinephrine levels and other fluctuating inputs, can be summarized by the heart rate variability. In other words, the variables whose salience may differ from part to part of a system can influence each other in interesting ways.

### Internal control functions of the heart

There are a number of internal control functions of the heart that allow it to process different inputs in different ways. We will describe some of them in detail in order to explain how the heart uses general rules governed by the following control functions, as part of its function as a biological computing system.

As an overview, the heart has internal pacemakers that can be compared to a clock or an old-fashioned metronome, but both of those metaphors are simplistic, because the heart’s natural pacemakers are flexible and responsive to the organism’s needs, both internally via stimulation within the heart and externally via input from the brain or hormones. It would be better to compare the heart’s pacemakers to a programmable metronome, to a film score’s tempo map, or to a conductor’s tempo setting. One indication that the heart’s natural pacemakers are more sophisticated than clocks (or non-programmable metronomes) is the fact that implantable artificial pacemakers have been programmable and reprogrammable since the 1970s.

First, one of the heart’s internal control functions is mechanical and increases stroke volume *SV*, the amount of blood ejected from each ventricle (now dropping the stroke number index *i* that was in the previous subsection), when ventricular filling is increased because of increased venous return. This is called the Frank–Starling law and is a result of the fact that cardiac muscle fibers have a typical resting length that is longer than the contracting length but quite a bit shorter than their optimal resting length, in the sense of being able to exert the most force and thus eject the largest volume. When venous return increases and the end-diastolic volume *EDV* increases, this causes a stretching of the muscle fibers to a longer resting length that is closer to optimal and thus allows the exertion of more force on contraction.

In notation (and ignoring right- and left-side differences), the Frank–Starling law gives stroke volume *SV* as a function of end-diastolic (fully filled) volume *EDV*, i.e., $SV = f(EDV)$, where *f* is a function called the Frank–Starling curve (see for example [15], p. 287). In healthy hearts, the Frank–Starling curve *f* is usually increasing, meaning that if the resting length of ventricular fibers is stretched to a longer resting length, then they exert a larger force when contracting. (In failing hearts, the curve *f* may actually be decreasing for larger values of *EDV*, meaning that the longest resting lengths are suboptimal.) The implication of this curve being increasing is that if venous return increases, then the volume of the blood arriving in the heart increases and stretches the muscle cells, so that the volume of the blood ejected from the heart increases also. The Frank–Starling curve can also be shifted upward under the influence of sympathetic stimulation, further increasing the maximal ejection volume. The healthy heart’s intrinsic ability to keep up with venous return is crucial to maintaining a well-distributed blood supply throughout the body (see also [16], p. 682).

Second, another one of the heart’s internal control functions is chemical, supplying heart muscle cells with increased amounts of nutrients through adenosine release and coronary artery dilation. The heart takes a large fraction (65%) of oxygen passing through the coronary system compared to the 25% that other tissues take from the bloodstream. Thus the heart does not have much oxygen reserve for activities like exercise and to counteract this must increase its own coronary circulation through the mechanism of dilating the coronary arteries. That mechanism is activated by the molecule adenosine (the same A as in ATP), which is released in significant quantities when heart muscle cells are working especially hard. Adenosine binds to receptors in the coronary arteries and dilates the vessels, thus allowing increased the blood flow to the heart muscle. This dilation is necessary because simply increasing the heart rate increases the amount of time that the open aortic valve blocks blood flow into the coronary artery, whereas adenosine increasing the diameter of the coronary arteries truly allows more blood to flow into the coronary circulation and supply the cardiac muscle cells ([15], p. 190, and [17]).

Third, another one of the heart’s internal control functions is electrical, helping to maintain a steady pace of pumping. This control function is governed by specialized muscle cells called auto-rhythmic cells that are distributed in two nodes (upper sinoatrial and lower atrioventricular node) and a ventricular network of bundles and Purkinje fibers. These auto-rhythmic cells encompass leading pacemaker cells in the sinoatrial (SA) node, latent pacemaker cells in the atrioventricular (AV) node, and even slower pacemaker cells in the Purkinje fibers, and they are all specially-adapted muscle cells that are weak in their contractile function but strong in their pacemaking function ([16], pp. 674ff).

The leading electrical pacemaker cells, in the sinoatrial node, have a set of baseline rates they operate at in the absence of external input (typically around 100 beats per minute). But the SA node’s pace can speed up or slow down in response to external input from the autonomic nervous system, which has two parts, sympathetic and parasympathetic. Thus the SA node’s average pace ranges between 70 and 100 beats per minute depending on the relative balance of sympathetic (tending to speed up) and parasympathetic (tending to slow down) stimulation.

The latent pacemaker cells, in the atrioventricular node, can act as a backup pacemaker if the SA node fails, although the AV node’s natural rate averages between 40 and 60 bpm, significantly slower so that it does not overtake the SA node under normal circumstance, and in fact about the right pace to maintain resting bodily functions but not high levels of activity such as exercise.

The even more latent pacemaker cells in the bundles and Purkinje fibers have two roles: (1) to act as a secondary backup if both nodes should fail, although their base rate is between 20 and 40 bpm, about enough to maintain the body sedentarily or in a coma, and (2) to have the possibility of overtaking both nodes in situations that might require a great deal of activity, in which case their excited rate is about 140 bpm, fast enough for fight or flight. (Common causes of this overtaking state are fear or anxiety and caffeine overstimulation.)

In addition to being part of the heart’s internal control functions, these auto-rhythmic pacemaker cells and all of the other electrically active muscle cells together form a biological vehicle that would be complicated to describe mathematically. A description would, for instance, require many coupled Hodgkin-Huxley-type differential equations, because the electrical activity consists primarily of waves of various ions passing through cardiac cell membranes and through gap junctions between cells. Next we will describe how these ion waves form a biological vehicle.

### Cardiac electrical activity as a biological vehicle

What follows is a detailed description of the ion flows in the heart, mostly at the cellular level, and then a discussion of some specific ways that this biological vehicle can be transformed.

The mechanics of the auto-rhythmic nodal cells is interesting, and can usefully be compared and contrasted with the mechanics of neurons firing. Both nodal cells and neurons have a negative resting membrane potential and they both fire upon reaching a threshold value. Other aspects of their functions are different. Nodal cells reach their threshold values at regular intervals because of a slow depolarization caused by a steady passage of Na$^+$ ions into the cell from the extra-cellular fluid (ECF), and by a deactivation of potassium channels that would otherwise allow K$^+$ ions to go out into the ECF. When enough ions accumulate in the cell and the membrane potential has reached its threshold, voltage-gated calcium channels are activated and there is a fast influx of Ca$^{++}$ ions into the cell, completely depolarizing it.

The abundance of Ca$^{++}$ ions inside the nodal cells causes more calcium release from the sarcoplasmic reticulum (SR) inside the cell, allowing the nodal cell’s few muscle fibers to contract—but more importantly, passing electrical current to neighboring contractile cells via gap junctions and causing them to experience their own action potentials and many of their muscle fibers to contract. The remainder of the nodal cells’ action potential is a repolarization caused by activation of potassium channels and a fast (but slower than neurons’) wave of K$^+$ ions leaving the cells. Then the K$^+$ channels are inactivated, the nodal cell membrane is briefly at rest, and then the steady leaking of calcium again slowly depolarizes the cell to start the cycle over again. The near-clock-work like effect of this process is affected by external input (sympathetic stimulation speeds the process up, and parasympathetic slows it down, through different mechanisms), but it’s remarkable that the nodal cells’ baseline pace-setting is innate and does not require external calibration.

The electrical activities of cardiac cells have interesting similarities and differences compared to the electrical activities of neurons and skeletal muscle cells. The fast depolarization of cardiac nodal cells is caused by Ca$^{++}$ influx, in contrast to the fast depolarization of neurons, skeletal muscle cells, and contractile cardiac cells all caused by Na$^+$ influx ([15], p. 83). Contractile cardiac muscle cells have a distinctive plateau shape for their action potentials, different from all other excitatory cells ([15], p. 272). This causes a refractory period that is much longer than neurons’ refractory period, and for the heart this is a mechanism to prevent muscle cell contractions that are too frequent (tetany) or uncoordinated (fibrillation), and hence ineffective for pumping.

These auto-rhythmic nodal cells provide a control mechanism over contractile cardiac muscle cells as follows. The abundance of calcium ions in the nodal cells’ cytoplasm passes through gap junctions to the surrounding contractile cells. This wave of positively charged ions depolarizes the contractile cells (much more quickly than the slow sodium leakage in nodal cells), which quickly hit their threshold and fire: the action potential of the contractile cells has its upswing caused by a fast influx of sodium ions through voltage-gated Na$$^+$$ channels. Then the sodium channels close and there is a plateau phase of the contractile action potential, which is very different from neurons’ and skeletal muscle cells’ action potential. This plateau is caused by a continued Ca$$^+$$$$^+$$ influx into the contractile sarcoplasm, which both slows the repolarization and provides a nontrivial fraction (10-20%) of the calcium ions that are needed for contraction of the cardiac muscle fibers. In particular, this Ca$$^{++}$$ influx stimulates T tubules and causes the sarcoplasmic reticulum (SR) to release even more Ca$$^{++}$$ (80-90% of the total calcium needed) into the sarcoplasm, and additionally, the abundance of positive ions (both sodium and calcium) creates a wave through gap junctions to other neighboring contractile cells so that they too can fire and contract. The speed of this electrical wave through the contractile tissue is fast compared to the slowness of the plateau and repolarization, an important feature that allows cardiac contractile tissue to contract in synchrony. The peak of contractile force is near the end of the plateau phase in the action potential, about 70 ms after the near-simultaneous myocardial depolarization for atrial muscle, and over 150 ms after depolarization for ventricular muscle. Repolarization is caused, as usual, by potassium channels opening and allowing K$$^+$$ ions to exit the cell rather quickly (though more slowly than Na$$^+$$ ions entered).

Near-simultaneous depolarization of the ventricular muscles is important for the coordinated pumping action of those chambers. Near-simultaneous depolarization of the entire heart muscle would be a problem, however, because efficient pumping requires the atria to contract before the ventricles. The nodal cell system has a mechanism for controlling this sequence of actions, too, as follows.

The heart has a fibrous skeleton connecting all of the valves and separating the atrial muscle tissue from the ventricular muscle tissue. Thus, the only electrical contact between the atria and the ventricles is at the second node of auto-rhythmic cells, the AV node. This node slows down the electrical wave passing through it, by 100 ms, so that the time that passes between full atrial contraction and full ventricular contraction is about 200 ms. This allows the coordination of contraction between atrial and ventricular chambers: atria must contract slightly before the ventricles do in order to completely fill the ventricles.

The above description of ion flows is a detailed way of understanding the heart’s electrical activity. But similar to the heart-rate and stroke-volume medical variables that summarize detailed features of the bloodstream vehicle, there are medical variables that summarize the detailed electrical activity, mainly the electrocardiogram (ECG). An ECG measures the electrical activity on the skin at 2 to 12 points where electrodes are attached to the body, so it provides only indirect measures of the heart’s activity (the result of electrical waves traveling from the heart through various tissues to the body’s surface) but it has the advantage of being non-invasive. And there is plenty of valuable data in ECG graphs, including about dangerous and benign variations in heart rhythms called arrhythmias.

How can this vehicle of electrical activity be transformed? In a number of ways, with internal or external causes. The next section will discuss some ways in which other organs can control the heart’s functions, and the rest of this section outlines two ways that the heart can transform its own electrical activity vehicle.

First, there is a special type of potassium channel that is sensitive to ATP levels, called the $K_{ATP}$ channel. Under typical circumstances, these channels in the cardiac muscle cells are closed, and the cell’s electrical current patterns proceed as outlined above. But if blood oxygen levels drop dramatically (called anoxia) or if blood is not flowing sufficiently to the cardiac muscle (called ischemia), then these $K_{ATP}$ channels open and change how the cardiac muscle cells’ electrical currents operate. Specifically, opened $K_{ATP}$ channels reduce the amount of calcium ions entering the cells and shorten the time between successive contractions [18]. This both speeds and weakens contractions, a situation that seems suboptimal but that actually protects the cardiac cells from dying, because calcium overload during ischemia is likely to damage cells. Thus this ATP-sensitive channel activation can help preserve cardiac tissue during a heart attack.

Second, a cool thing that a heart can do is restart its own electrical activity after it has been transplanted into another person. This is a healthy and common response to the chambers refilling with blood after the bypass machine is removed. Sometimes an electrical shock is needed to restart a transplanted heart, but spontaneous restarting happens much of the time.

### External control functions on the heart

The body also has a number of mechanisms external to the heart that control aspects of heart function. These external control functions can be viewed as producing input instructions or input data to the heart depending on bodily states and activity, for instance. Such instructions are delivered through various vehicles (mainly in blood particles and nerve impulses) after being produced by other bodily tissues such as the autonomic nervous system and the adrenal glands.

There are several ways that the autonomic nervous system (ANS) changes heart function, specifically stroke volume and heart rate, two examples here for the sympathetic (SNS) branch of the ANS and a third example for the parasympathetic (PNS).

First, the sympathetic nervous system conducts signals to the heart muscle cells that increase their contractility by increasing calcium ion flow into the cells and thus increasing the strength of contraction and speeding relaxation of the fibers by increasing calcium movement in both directions, into and out of the sarcoplasm. This results in the increased movement of the muscle fibers and the entire muscle producing more force and thus ejecting a larger fraction of blood from the ventricles, another important medical variable called the ejection fraction. Even in the absence of increased filling of the chambers (and the resultant benefits of an increasing Frank–Starling curve), the increased ejection fraction increases stroke volume.

Second, an increase in SNS activity also increases heart rate by increasing the concentration of norepinephrine, which binds to $\beta _1$-adrenergic receptors on autorhythmic nodal cell membranes and which changes ion channel activity in such a way as to cause the threshold potential to be reached more quickly [16]. This has the effect of increasing the heart rate.

These two effects, increasing both contraction efficiency and heart rate, combine in an important way so that increased SNS (“fight or flight”) activity increases overall cardiac output (CO) and thus the body’s ability to respond to a threat or to exercise. The reason is that simply increasing the heart rate but not contraction efficiency could reduce the stroke volume and possibly even the overall cardiac output.

Third, the PNS (parasympathetic nervous system) can exert control on the heart rate by releasing acetylcholine, which opens potassium channels and thus lowers the resting potential of the autorhythmic nodal cells. This lengthens the time until the sodium ion drift causes the membrane potential to reach threshold, lowering the heart rate. The PNS has essentially no effect on contractility, because vagal innervation is concentrated on the nodes and is sparse on the ventricles [16].

There are several ways that the endocrine system exerts influence on heart function as well, also affecting cardiac output (CO) in ways that are crucial to bodily function.

One of the hormones increasing cardiac output is epinephrine. Epinephrine is synthesized mainly by the adrenal cortex, which increases Ca$^{++}$ movement and fiber contractility, and this hormone is transported to the heart in the bloodstream, a slower vehicle than the SNS electrical activity that releases norepinephrine directly onto cardiac cells. Both norepinephrine and epinephrine increase both contractility and heart rate, thereby increasing CO.

Another one of the hormones is thyroxine, which arrives at the heart through the coronary bloodstream from the thyroid, and causes lasting increases in heart rate. Thyroxine also modulates the effects of norepinephrine and epinephrine on the heart, enhancing their effect. So thyroxine can be seen in this way to alter the internal state of the heart, which change the rule according to which the heart responds to other hormonal input.

(In technical terms, sympathetic stimulation and epinephrine both shift the Frank–Starling curve upward, which means that ventricular fibers can exert even more force than when they are stretched by increased chamber filling, as in the above internal mechanical control mechanism. So the precise balances of SNS stimulation and hormone concentrations can be viewed as internal states of the heart or internal states of the body, as appropriate.)

Another external control mechanism that is neither hormonal nor autonomic is ion concentrations in the bloodstream and extra-cellular fluid. These balances of electrolytes influence the activity of the heart, mainly through imbalances in the normal plasma electrolyte levels. For instance, high potassium levels can cause the heart to stop by depolarizing the nodal cells beyond a level for which their sodium ion drift can reach threshold. Low potassium, on the other hand, can cause weak contractions and arrhythmias. Low calcium can depress the heart; high calcium increases heart rate and contractility; and very high calcium can disrupt the heart by causing dangerous arrhythmias. For reasons including these, the concentrations of certain ions are typically tightly controlled by the body, for example, calcium by the hormones parathyroid and calcitonin.

### Internal states of the heart

A complete description of the internal state of a heart would be unwieldy, requiring exact description of the entire organ (and some other parts of the body) down to the molecular level. But many of these exact microstates are sufficiently similar to each other to be aggregated into the same macrostate. And certain measurable variables for the person have strong enough correlations with their heart function that their heart’s internal state can be roughly described by an array of numerical values for these variables.

Examples of such variables include age, gender, exercise habits, and body temperature. These are fairly high-level variables and may correspond to fine-grained descriptions such as gene expression, hormone levels, and electrolyte and energy status at the cellular and molecular levels. Additionally, mid-level transient variables, including the medical variables such as heart rate, stroke volume, ejection fraction, cardiac output, blood electrolytes, and electrical traces, all give some indications about the details of the heart’s internal state.

These internal states can be quite persistent and stable until a change happens that is significant enough to move it to a new internal state that is similarly stable: this modest and modifiable stability could be formalized as an example of metastability. Metastability in general, and the heart’s internal state in particular, are persistent across longer times-cales such as months, allowing the storage of biological states as a kind of memory. One effect of aging on the heart is that some calcium channels are lost, reducing the heart’s responsiveness to sympathetic stimulation by norepinephrine. This can be viewed as habituation, a non-associative form of learning ([15], p. 288).

Exercise, especially endurance exercise, can remodel heart tissue. Heart enlargement has been observed among athletes since 1899, but this differs from the heart enlargement that occurs with disease: a diseased enlarged heart usually has a thinner wall of the left ventricle (LV), whereas an exercise-adapted enlarged heart typically has a thicker LV wall. The athlete’s larger and thicker-walled left ventricle has both increased end-diastolic volume and a slightly decreased end-stroke volume compared to the volumes prior to athletic training, a result of increased hemo-dynamic stress. The outcome of these volume changes is to increase stroke volume, sometimes dramatically, which when combined with increased heart rate can raise cardiac output by a factor of about 5 or 6, compared to the factor of 2 or 3 that would result from increased heart rate alone [19]. This training-induced cardiac remodeling lowers the energy requirements for future exercise and enhances the person’s performance ability. It is a form of memory held physically in the heart, which can take quite a long time to lose.

A mid-level description of the heart is also persistent across shorter time-scales. On time-scales such as days, an example is the scar tissue that may develop after a heart attack, influencing the function of the heart, possibly permanently. Specifically, the scar tissue may change the responsiveness of a region of contractile cells to the electrical signals of the pacemaker cells. Another example is on short time-scales such as seconds, where there is a persistence of cardiac rhythms, summarized in the maxim of cardiology, “Fibrillation begets fibrillation; sinus rhythm begets sinus rhythm.”

### Summary of the heart as a biological computer

The heart can be viewed as a biological computing system because it is a biological mechanism whose purpose is partly to manipulate the biological vehicle that is the bloodstream according to rules outlined above including the internal control functions, which are more numerous than presented here. These rules also demonstrate how the heart is sensitive to biological distinctions, and the thresholds for those distinctions usually depend on the specific biological particles in question.

The heart has other purposes which also fall under the framework of biological computation, including sending output to other parts of the body as a result of its internal state and which result in modifications to other bodily processes. One example is the production of the hormone Atrial natriuretic peptide (ANP) in response to, for example, the physical stretching of the atrial chambers, and which is carried by the bloodstream to signal the kidneys to excrete sodium and lower blood pressure. This peptide also has a paracrine (local endocrine) function to cause changes in the heart’s own muscle tissue that can prevent hypertrophy, a potentially undesirable adaptation to the increased chamber filling called volume overload.

The heart can manipulate its biological vehicle of electrical activity, in addition to receiving input from the nervous system to modify this vehicle. Similar to the bloodstream being a medium-flexible vehicle as discussed in Sect. 2.1, electrical activity is also medium-flexible. Different species have different variations on the exact structures of the ion channels and the other proteins involved. And dysfunctional aspects of heart electrical activity can be supplied by technological advances, prominent examples being the programmable pacemaker and the implantable defibrillator.

## The venus flytrap: *Dionaea muscipula*

*Dionaea muscipula* can also be viewed as a biological computing system whose purpose is partly to trap and digest potential food without wasting energy in disadvantageous circumstances. The main vehicles for Dionaea are the sequences of objects that may enter its traps (some of which may be non-biological), and the traps’ internal electrical activity (similar to the heart and not involving any neurons).

### Description of Dionaea’s trap algorithms

The tissue in Dionaea’s traps is electrically active under mechanical stimulation, with varying outcomes depending on internal and environmental conditions for the plant. Each of the two lobes of a Dionaea trap has about three hairs, and these hairs are sensory which allow the organism to measure the number and length of appropriate stimulations delivered to the trap: repeated mechanical stimulations cause depolarization, an accumulation of electrical charge, and once the depolarization reaches an electrical threshold value, the tissue produces an action potential that causes the closure of the trap’s two lobes (details to follow). The electrical threshold varies based on the size of the trap: for instance, the threshold is lower for smaller traps and higher for larger ones.

The algorithm that Dionaea appears to use for closing a trap combines internal control mechanisms with internal states influenced by environmental factors and goes as follows:

- If the ambient environment is humid and warm (28 to 36 degrees Celsius), then one mechanical prey stimulation of a trap hair is sufficient for trap closure [20].
- If the ambient temperature is humid and moderate (around 22 degrees Celsius), then one mechanical stimulation is insufficient and two stimulations within a short enough time frame, about 20 to 30 seconds, are sufficient for closure of the trap [20].
- If the ambient environment is dry and either moderate or warm, then two mechanical stimulations are insufficient and three stimulations (again within a short enough time frame) are sufficient for trap closure ([21], p. 182).

The reason for this algorithm is probably the plant’s need to conserve energy. Trap closure consumes energy, and each trap can survive through about a dozen closures only, so the benefits of each closure should be worthwhile: closing on a raindrop or a stone would be wasteful, but insect prey is likely to stimulate trap hairs multiple times. Another aspect of conserving energy is to avoid unnecessary digestion because it is energetically expensive, so there is a second algorithm that the plant seems to use for starting digestion, below.

Trap closure is very fast, starting about 0.1 to 0.2 seconds after the threshold stimulation is reached and the action potential is started, and finishing within half a second [21]. The trap can be manipulated by experimentalists to close without mechanically stimulating the trap hairs but instead electrically stimulating a small region near the midrib between the two lobes, where motor cells are thought to be. These experiments have shown that a number of small subthreshold electrical charges can have a cumulative effect reaching the threshold value and causing trap closure [22].

After closing a trap, Dionaea’s algorithm for selecting objects to digest is:

- If the prey is small enough, then it may escape through small gaps between the trap’s interlocking teeth, and the trap will later re-open without having performed the digestion process ([21], p. 183).
- If the prey is too large to escape, then its movements continue to stimulate the mechano-sensory hairs, and this generates more action potentials that lead to the tighter closure of the trap and hermetic sealing before digestion.

How are these sophisticated algorithms accomplished by Dionaea? It turns out that the traps’ trigger hairs work similarly to the hairs in animals’ inner ears (see e.g., [15], p. 182ff). At the base of a Dionaea trigger hair, there is a ring of mechano-sensory cells surrounding the place where the hair bends most of all. The bending of the hair deforms the sensory cells, putting pressure on certain intracellular complexes (ER-vacuoles) that release ions, and opening mechanically the membrane ion channels that allow the cells to depolarize ([21], p. 181).

Subsequent bending of the trigger hairs cause further depolarization, and when a certain threshold is reached, voltage-gated ion channels open and allow fluxes of calcium, chloride, and potassium ions in an action potential that spreads evenly throughout the tissue and causes the trap to close, quickly at first and then slowly at the end, similar overall to a soft-close toilet lid. The quick part of the closure process appears to be governed by a combination of hydraulic action and elastic instabilities due to the geometric curvature inversion of the lobe from concave to convex, though this process is not yet fully understood ([21], p. 183).

Both trigger-hair mechano-sensory cells and inner ear hair cells are examples of mechano-transduction that generate depolarization called a *receptor potential*, a subthreshold potential that may precede an action potential. In the inner ear, a hair cell’s receptor potential causes a release of neurotransmitters and an action potential in a neighboring cell. In Dionaea by contrast, the mechano-sensory cells’ receptor potential causes instead an action potential in the same cells, an action potential that is conveyed through the tissue by small gap-junction-like holes called plasmodesmata. The plasmodesmata are tiny channels across the cell walls that connect the cytoplasm of neighboring cells and that enable the passage of small particles from one cell’s cytoplasm to the next.

(In fact, these plasmodesmata channels are similar to the gap junctions between cardiac muscle cells: Both plasmodesmata and gap junctions play a similar role in electrical transmission by allowing waves of ions to flow from cell to cell in the tissue.)

The remaining part of the trap closure (the slow part) is caused by further action potentials from the prey’s struggles stimulating the trigger hairs, and by inner trap cells sensing nitrogen-rich substances such as urea from the prey ([21], p. 183). The trap responds to these stimuli by sealing off a chamber between the two lobes for digestion.

Finally, we briefly describe the digestion process in a closed Dionaea trap. The tissues of prey are rich in a variety of nutrients: chitin, lipids, proteins, nucleic acids, and carbohydrates, so the trap tissues produce a similar variety of enzymes to break these tissues down into usable components: two chitinases, a lipid transfer protein, five proteases, four nucleases including both DNase and RNase, three carbohydrate-hydrolyzing enzymes, and additionally, two phosophatases ([21], p. 208f). From this array of enzymes, the Dionaea trap digestive compartment selects and releases a combination of enzymes that is suitable to the particular prey in the trap. In addition to enzymes, Dionaea’s digestive fluid also contains a wide array of ions, especially the H$^+$ particles from hydrochloric acid regulated by proton-pump ATPases in the cell membranes. This optimizes the function of many of the enzymes, which work best at an acidic pH.

Dionaea uses electrical signals for the digestion process as well as the closure process: electrical signals stimulate the approximately 30,000 small digestive glands on the inner surfaces of the trap lobes. Dionaea has the ability, similar to the stimulation-counting described above for trapping the prey, to measure how much the trapped prey is struggling and to respond appropriately based on how many action potentials have been produced:

- After two action potentials, intracellular calcium levels increase and the plant hormone jasmonic acid (JA) is released to activate a signaling pathway.
- After three action potentials, genes for digestive enzymes are expressed.
- After five action potentials, nutrient- and sodium-transporters are activated that can identify whether the prey is sodium-rich and nutrient-rich [23].

A positive feedback loop also happens, where breakdown of prey tissues further stimulates the jasmonic acid (JA) signaling pathway in two ways: (1) potassium ions depolarize the glands’ membrane potentials which then stimulate the JA pathway, and (2) chitin molecules directly stimulate the JA pathway. Stimulating the JA signaling pathway causes the release of more digestive enzymes, a feedback loop that ends when the prey has been entirely digested.

Afterward, the trap re-opens gradually over a period of one or two days, by growth processes and hydrostatic pressure adjustments, the details of which are not fully known, processes that limit the closure of the trap and the use of it as a digestion chamber to about a dozen cycles [21].

The upshot of all of this is that Dionaea is sensitive to differences in the vehicle of the sequence of input objects, the small animals or debris that land between the two lobes, and it treats distinguishable vehicle portions appropriately with respect to their differences along a few axes: digestible or not, nutrient-rich or not, repeatedly moving or not. One could describe the potentially stimulating object similar to the bloodstream vehicle, perhaps listing all of the sub-components of the object as a vector again, but the plant’s response depends primarily on these few axes.

To give some realistic examples: (1) A small stone is not digestible, not nutrient-rich, and not moving after it comes to rest, so the trap would be unlikely to close in the first place (unless in warm conditions), would probably not finish sealing off the chamber, and almost surely would not go to the effort of releasing digestive enzymes. This way energy is not wasted trying to digest the stone. (2) A piece of organic debris would probably not stimulate trap closure by itself, but if it were accompanied by a suitable prey animal and became trapped together, Dionaea’s digestive enzymes would be able to digest the organic debris in addition to the animal. (3) A very tiny prey animal may stimulate the closure of the trap, but then escape and not stimulate the expensive digestion process.

And to give some thought-experiment examples: A robotic fly would probably trigger closure and perhaps start the digestion process, but then the digestion process would not fully ramp up (assuming the robot lacks potassium and chitin), and Dionaea would save energy by not producing further digestion enzymes. If the robotic fly contained nutrients, that would be a different story of course.

Taking a step back, we often think of plants as passive occupants of their environments, but in fact plants can effect changes on their surroundings, and Dionaea does so in a striking way. Dionaea may not be able to control what comes into its trap, but it does release compounds that are detectable by its preferred preys’ olfactory systems, especially Drosophila. These compounds include terpenes and benzenoids, a combination that appears to mimic Drosophila’s main food source scents, fruit and flower scents [24]. In addition, Dionaea produces visual signals to attract its preferred prey, UV-wavelength and fluorescent signals ([21], p. 286).

### Dionaea as a biological computing system

These behaviors, from prey-attracting signals to selective closure and a self-limiting digestion process, together form a sophisticated mechanism for Dionaea to obtain the nutrients that it needs without wasting energy on non-nutrient-rich objects. The counting algorithms for closure, sealing, and enzyme release and the feedback mechanism for digesting the prey efficiently, form a computational system that manipulates medium-flexible vehicles according to a general rule that also depends on the plant’s internal states. Roughly speaking, the general rule is “digest the food present; don’t digest non-nutrient-rich items,” a rule based on underlying biological demands that encourage organisms to optimize energy and nutrition in their environments.

Dionaea’s internal states include adaptations to temperature and water supply, and these internal states influence which part of the general rule is implemented. At a lower temperature, fleeting or small prey animals are likely to be ignored; but at a higher temperature, they may be trapped. Under drought conditions, small prey is even more likely to be ignored in favor of large prey that lingers on the lobes of the trap, making the costly digestion process more likely to be worthwhile for the plant, which is already under stress.

And how does Dionaea use the nutrients that it acquires? A large amount of the digested carbon-containing molecules are used in the plant’s respiration process by about two days after feeding and released into the atmosphere as CO$_2$. Additionally, molecules acquired from prey digestion provide building blocks for the plant’s cellular structures and processes. Most of the prey-derived amino acids appear to be retained by the trap doing the digestion, with small amounts going to the plant’s other traps and roots. In particular, about 5 weeks after feeding, the plant has a higher capacity for photosynthesis [25].

The general rule that Dionaea uses is an analog-to-digital one. The input vehicle (prey animal, debris, etc.) has continuous location and velocity as functions of (continuous) time, and these continuous variables can be discretized by the discrete locations of the sensory hairs and the discrete counting that the traps appear to do. The digestion process releases a discrete number of enzymes, breaking the prey tissues down into discrete organic molecules (sugars, amino acids, nucleobases, lipids, vitamins, phosphates) for use by the plant. These output molecules may have continuous locations and velocities as a function of time, so they may be considered technically continuous, although a discrete description of their role in the plant’s cellular processes may approximate those continuous quantities well enough for the two descriptions to be biologically indistinguishable.

To see the medium flexibility of the input vehicle, note that any suitably-sized object of almost any medium can be placed into Dionaea’s trap, and the trap will process it according to the rule above, meaning that it extracts needed nutrients from nutrient-rich moving objects and that it does not waste too much energy on non-moving or non-nutrient-rich objects. This is not a binary rule: for instance, whether a leaf fragment is digested depends on whether it co-occurs with moving prey, and whether a tiny prey animal is digested depends on whether it escapes or not, which may be a random event. This may be closer to medium-independence than the bloodstream, because almost any object can be processed according to the trap’s rule, with the exception of a few potential objects that could harm the plant and thus prevent its usual behavior, such as a droplet of acid strong enough to damage the trap.

The medium flexibility of the electrical activity vehicle in the trap lobes, similar to the electrical activity in the heart’s muscle tissues, is more clearly not medium-independent. In both cases, particular ions play particular biological roles, and these roles cannot be switched even for similar ions. For instance, sodium and potassium ions are chemically similar, both having a similar ionization energy and a resulting +1 charge, but Na$^+$ and K$^+$ are biologically distinguishable, having sufficiently different radii that ion channel proteins can play their gatekeeping roles. And yet, there remains some medium flexibility, such as the three common isotopes of potassium playing the same role in the organism with negligible biological distinction.

In summary, Dionaea has molecular processes for implementing these algorithms, with conditional branching governed by its internal states (which are in turn influenced by external conditions), upon the vehicles of trap input and trap electrical activity. The fact that Dionaea’s algorithms can adapt to different environmental conditions illustrate that the plant has molecular processes that change its electrical activity thresholds, even if scientists do not fully understand the molecular mechanisms underlying trap function.

## Conclusion

Analyzing how hearts and flytraps can do both biophysical and computational actions is important for clarifying how biological computation is similar to–and different from–physical computation. A main point of this work has been to demonstrate that there are significant overlaps between natural accounts of physical computation and biological computation, and there are subtle distinctions that may help us get to a better understanding of all computing machinery and systems, both technological and biological.
